# Prevalence of food thermometers usage and temperature control in restaurants in Dammam, Saudi Arabia

**DOI:** 10.1002/fsn3.3305

**Published:** 2023-03-10

**Authors:** Nargis Begum Javed, Mohammed AL‐Mohaithef

**Affiliations:** ^1^ Department of Public Health, College of Health Sciences Saudi Electronic University Dammam Saudi Arabia; ^2^ Department of Public Health, College of Health Sciences Saudi Electronic University Riyadh Saudi Arabia

**Keywords:** documentation, Food handlers, freezer, monitoring, refrigerator, survey

## Abstract

The Saudi Ministry of Municipal and Rural Affairs is planning to initiate a hazard analysis critical control point (HACCP) system in restaurants and cafeterias to manage issues of food safety in Saudi Arabia. One of the most important elements in the HACCP system is the monitoring temperature of cooked and stored food. The present study aimed to investigate the prevalence and use of refrigerators/freezers and food thermometers among food handlers in local and international restaurants in Dammam, Saudi Arabia. A cross‐sectional study was conducted in municipality‐licensed restaurants. The temperatures of refrigerator and freezer were checked, and the survey form was completed by the researcher according to logbook entries. We then checked for the presence of a food thermometer, and if a functional thermometer was present, the chef was asked to complete an online questionnaire using Survey Monkey website on a tablet. The response rate of the survey was 68% (238/350 restaurants). We found that 88.1% of restaurants used a thermometer to check the temperature of their refrigerators and freezers. Thirty‐one restaurants (13.0%) had a regular temperature‐monitoring record available for both the refrigerator and freezer. International restaurants had significantly greater temperature‐monitoring data than local restaurants (88.1% vs 63.3%; *p* = .0001). The prevalence of food thermometers in restaurants was 53.4% (127/238 restaurants), with significantly higher prevalence in international restaurants than in local restaurants (96.6% vs 10.8%; *p* = .0001). The practice of using food thermometer “always” when meat turned brown was significantly associated with the chef's age and education level. The study results showed poor monitoring and documentation of refrigerator and freezer temperatures along with a low prevalence of food thermometer use. The study result provides an insight into one of the barriers to the implementation of the HACCP system in Dammam.

## Abstract 2

The research is of Public Health Importance as it deals with the temperature‐monitoring practice followed by restaurants. Temperature monitoring is one of the principles of HACCP, which can help to reduce the risk of foodborne illness.

## 1 INTRODUCTION

Foodborne diseases (FBDs) are a major public health issue worldwide, and Saudi Arabia is no exception. A study conducted in Qassim, Saudi Arabia, reported that 68.9% of FBDs were due to consuming food prepared in food service establishments such as restaurants, canteens, and fast food outlets (Al‐Goblan & Jahan, 2010). Another study used the information available from the Ministry of Health in Saudi Arabia Statistics and reported that FBD outbreaks increased from 2010 to 2011 and that food service establishments were responsible for 62% of these outbreaks (Al‐Mutairi et al., 2015). Studies from Saudi Arabia reported foodborne outbreaks to be linked with *Salmonella* spp., nontyphoidal *Salmonella* spp., *Staphylococcus aureus* (*S. aureus*), *Shigella* spp., *Camphylobacter jejuni* (*C. jejuni*), *Bacillus cereus* (*B.cereus*), *Clostridium perfringens* (*C. perfringens*) (Al‐Goblan & Jahan, 2010; Al‐Joudi, 2007; AlMaghderi & AlMazroa, 2003; Al‐Saydalani, 2011; Alsayeqh, 2015a).

*Salmonella* spp., *Escherichia coli* (*E. coli*), and *S. aureus*, which are not able to multiple at temperatures below 5°C, may benefit from inadequate control of refrigeration temperature and start multiplying causing FBDs if consumed. Some bacterial species, including *Clostridium botulinum* (*C. botulinum*), *B. cereus*, *C. perfringens*, and *Campylobacter* spp. may not grow at temperatures below 5°C but may survive at these temperatures, and when the refrigerator temperature exceeds 10°C, it can start multiplying (Kassianenko et al., 2005). According to Centers for Disease Control and Prevention (CDC), the four steps to food safety include cleaning, separating, cooking, and chilling. Bacteria can multiply rapidly in food if left in the “Danger Zone” between 4°C and 60°C and the CDC recommends to keep the temperature below 4°C for refrigerator and below −18°C for freezer (CDC, n.d.) (https://www.cdc.gov/foodsafety/keep‐food‐safe.html). US Department of Agriculture (USDA) reports that foodborne illness caused by *C. perfringens* and *S. aureus* can be prevented by storing hot food hot and cold food cold [below 4°C] (USDA, n.d.) (https://www.fsis.usda.gov/food‐safety/foodborne‐illness‐and‐disease). The increase in the refrigerator temperature can result in an increase in bacterial growth, especially in time temperature control safety (TCS) food such as cheese, yogurt, meat, salad dressings, and dishes containing eggs.

Studies have reported that undercooked meat is also responsible for FBDs due to pathogens such as *Salmonella* serotypes, *E. coli*, and *C. jejuni* (Al‐Mutairi et al., 2015; Boore et al., 2010; USDA, n.d., https://www.fsis.usda.gov/food‐safety/foodborne‐illness‐and‐disease). Consumers and chefs usually use a number of ways to determine whether food is adequately cooked, such as the color of meat, which is often used as an indicator of appropriate cooking (Lin, 2018). However, studies have reported that color of meat is not a reliable indicator of internal temperature and destruction of bacteria (Bergsma et al., 2007; Hague et al., 1994). Other methods that are used to confirm that meat is cooked include testing firmness, the color of juices, and loose joints, although most of these methods are not correlated with microbiological safety (Berry et al., 2001). The most reliable method to verify food is adequately cooked is measuring its internal temperature with a food thermometer. Research studies have reported that the use of cooking thermometers to check internal temperature of cooked food is crucial to prevent FBDs caused by pathogens such as *C. jejuni*, *Salmonella spp*., *E. coli O157:H7*, *Toxoplasma gondii*, *Yersinia enterocolitica*, *B. cereus*, *C. perfringens*, and *S. aureus* (https://www.cdc.gov/foodsafety/keep‐food‐safe.html, CDC, n.d.; Hillers et al., 2003; Medeiros, Kendall, et al., 2001; Medeiros, Hillers, et al., 2001; USDA, n.d., https://www.fsis.usda.gov/food‐safety/foodborne‐illness‐and‐disease).

In Saudi Arabia, two government organizations, Ministry of Municipality and Rural Affairs (MOMRA), 2014 and Saudi Food and Drug Authority (SFDA), n.d., play major roles in the management of food safety. MOMRA has issued a document with requirements that need to be fulfilled when opening a restaurant (https://amanatalbaha.gov.sa/main/ViewPDF.aspx?file=8ecd06bc‐71db‐4d13‐a540‐34126c607896.pdf, 2014). The document includes general requirements (infrastructure details of restaurant and equipment), special requirement (food transportation, storage, preparation, and general hygiene and inspection records), and personal categories (health certificates and appearance and behavior). All the foods used to prepare meals must be fresh and clean, free from signs of damage and corruption, suitable for human consumption and conform to the Saudi standards for each type. Foodstuffs must be stored at the appropriate temperatures for each type by cooling or freezing or at room temperature (https://amanatalbaha.gov.sa/main/ViewPDF.aspx?file=8ecd06bc‐71db‐4d13‐a540‐34126c607896.pdf, 2014). SFDA cold storage and safety of meat requirement guidelines are maximum storage temperature for chilled meat (1 ± 0.05)°C, frozen meat (−18°C), fluctuation of the temperature inside the refrigerator or freezer must not be higher or lower than 2 degrees Celsius and meat must be chilled or frozen at quantities within the storage capacity of refrigerating machine, which must have a thermometer and temperature recording equipment (https://www.sfda.gov.sa/sites/default/files/2021‐04/ColdStorageandSafetyofMeat.pdf , n.d.). The food safety infarction and fraud observed in Saudi Arabia related to temperature control include improper food handling and insufficient cold holding practices/equipment and fines for improper storage (refrigerator at <4°C or the freezer <−18°C) is $266 (Alsayeqh, 2015b; https://balady.gov.sa/Services/DownloadAttachment/2., n.d.).

Hazard Analysis Critical Control Point (HACCP) preventive measures are applied in Saudi Arabia for inspection of imported foods (Alsubaie & Berekaa, 2020), and SFDA is planning to make HACCP implementation mandatory for restaurant in the near future. The HACCP system is a systematic methodology for creating a food safety program to reduce the risk of FBDs in food establishments by focusing on various steps of the food preparation process, which starts when the raw products are received and ends with the final product being served (U.S. Food & Drug Administration, 2006). The HACCP system lists seven principles, which include hazard analysis, critical control point (CCP) identification, establishing critical limits, monitoring procedures, corrective actions, verification procedures, and record‐keeping and documentation (U.S. Food & Drug Administration, 2006).

The lifestyle in Saudi Arabia has changed in past decades; restaurants and cafes have become one of the hotspot for socializing and interacting with friends and relatives. Improper temperature control in restaurants can put consumers at risk of FBDs. The implementation of the HACCP principles is presently not mandatory for restaurants in Saudi Arabia, but self‐adoption of monitoring and documenting refrigerator and freezer temperatures by the restaurant is considered a good practice. Moreover, effective documentation of temperature monitoring will help restaurant owners prove their due diligence.

To date, there is a lack of information regarding the use of refrigerators and food thermometers in restaurants, which is important in implementation of the HACCP system in Saudi Arabia. Therefore, the present study aims to investigate refrigerator and food thermometer' prevalence and its practice among food handlers in local and international restaurants of Dammam.

### 1.1 Research questions

1. What is the prevalence of built‐in‐thermometer refrigerators and freezers in the restaurants of Dammam? 2. Do restaurants use thermometer to monitor and record the refrigerator and freezer temperatures in Dammam? 3. What is the prevalence and type of food thermometer available in restaurants of Dammam? 4. What is the practice followed by the chefs in the restaurants having food thermometer? 5. Does the demographic profile of chef be associated with his practice of food thermometer usage?

## 2 MATERIALS AND METHODS

### 2.1 Study design

A cross‐sectional study design was used to evaluate the maintenance of refrigerators and freezers along with the prevalence and use of food thermometers in local and international restaurants. International restaurants serve international cuisines, mainly international food brands, such as fast food and burger restaurants, pizzerias, Indian, Pakistani and Chinese food, while local restaurants are restaurants that serve local Saudi Arabian cuisines. The inclusion criteria were restaurants must have a food license issued by the municipal council, located in Dammam city. Dammam was selected for this study because it is one of the largest industrial and tourism cities and the capital of Eastern Province, Saudi Arabia. It is the sixth populous city of Saudi Arabia, spread over an area of 800 km^2^ with a population of approximately 1.2 million. Many national and international companies are based in Dammam, leading to diversity in local and international restaurants.

### 2.2 Recruitment and data collection

A list of total of 350 registered/licensed restaurants was provided by the municipality of Dammam. All the restaurants were visited after obtaining approval from the MOMRA. The researcher showed the permission letter issued by the Ministry to the restaurant supervisor, and if he agreed to participate, the researcher reviewed the temperature of the refrigerator and freezer (if the restaurant had multiple refrigerators or freezers, we randomly selected one of each for review) using his thermometer and completed the survey according to the restaurant's refrigerator and freezer maintenance practice based on the logbook entries (checklist is available in Appendix S1). We then looked for the presence of a food thermometer and provided the chef with the online questionnaire using Survey Monkey on a tablet for self‐administration under the supervision of the researcher. The questionnaire had two sections: the first one had four questions on demographics, and the second section had five questions on the use of food thermometers. The questionnaire was finalized by external experts in the field before being used, and the questionnaire used is available in Appendix S1. The visits were without notification to the restaurant supervisors.

### 2.3 Data analysis

The statistical package software for social sciences (IBM SPSS Statistics Version 24) was used for the analysis. Descriptive analysis was conducted for all the variables (independent and dependent) and is reported as frequency and percentage. The comparison was performed between the refrigerators/freezers with built‐in thermometer (independent variable) and the monitoring of refrigerators/freezers temperature (dependent variable), followed by the comparison of type of restaurant (independent variable) with the presence of built‐in thermometer refrigerators/freezers and its monitoring practices (dependent variable). Furthermore, a comparison was performed between the independent variables (type of restaurant and age, education, and work experience of chef) and dependent variables (responses of chefs to food thermometer practice questions). The chi‐squared test was used for comparison, and a *p*‐value of <.05 was considered significant.

### 2.4 Ethical considerations

The study protocol was reviewed and approved by the Institutional Review Board of the Saudi Electronic University. The restaurant supervisors were briefly debriefed about the purpose of the research and the confidentiality of their responses. They were informed of the voluntary nature of their participation in the study and of their right to refuse to participate. After obtaining informed oral consent, we proceeded with the examination of the refrigerator, freezer, and food thermometer along with the logbook, and we provided the online questionnaire to the chefs to complete it.

## 3 RESULTS AND DISCUSSION

Of a total of 350 restaurants visited and invited to participate in the study, 238 (68.0%) restaurants agreed to participate and allowed the researcher to look at and review the condition of the refrigerator, freezer, and food thermometer used in the restaurant. Among the 238 refrigerators and freezers reviewed 177 (74.4%) refrigerator and 163 (68.5%) freezers had built‐in thermometers, reflecting that a higher percentage of the restaurants had refrigerators and freezers (appliances will be used when refrigerator and freezer are addressed together) with advanced technology. The researcher measured the temperature of the appliances using his thermometer and found that variance of readings between the displayed and recorded temperature was ±0.3°C. The mean ± SD of the recorded temperature of refrigerator and freezer were 3.4 ± 0.5°C (2.5°C to 4.2°C) and − 20.1 ± 1.1°C (−22.5°C to −18.3°C), respectively. The presence of appliances with built‐in thermometer can be both a boon and bane, as food handlers can monitor the temperature of the appliance without extra efforts, but the bane associated with this is that the temperature reflected may not always be accurate and food handlers may become negligent toward regular monitoring of the appliances with thermometers. In our study, the temperature of all the refrigerators and freezers examined were maintained within the SFDA‐recommended temperature range. A study by Brown et al. (2018) reported that 17.1% of delis in their study had at least one refrigerator temperature above 5°C, and 15% did not calibrate the refrigerator thermometer. Furthermore, they reported that delis, where refrigerator temperatures were not recorded by the staff, had a greater probability of the refrigerator being at a temperature higher than 5°C than those in which staff recorded refrigerator temperatures (Brown et al., 2018).

### 3.1 Practice of monitoring appliances temperature using thermometer

A comparison of the practice of monitoring appliances temperatures according to the presence or absence of a built‐in thermometer is shown in Table 1. The practice of maintaining the monitoring records of the appliance temperature was significantly higher in the restaurants that had built‐in thermometer appliances than in those with appliances without built‐in thermometers (*p* = .0001), while the practice of monitoring and recording the temperature “always” was higher in restaurants with appliances without built‐in thermometers than in those with built‐in thermometer appliances (19.7% vs. 10.7% for refrigerator and 14.7% vs. 12.3% for freezer) (Table 1). The finding suggests that training in temperature control is required for food handlers to understand the importance of monitoring the appliance temperature. The training should highlight the fact that most of the restaurant food product loss and food safety issues are associated with temperature or time controls.

**TABLE 1:** Comparison of practice of monitoring refrigerator and freezer temperatures according to the presence or absence of an inbuilt thermometer (*n* = 238 restaurants).

	Inbuilt thermometer in refrigerator		*p*‐value
	Present (*n* = 177)	Absent (*n* = 61)	
The thermometer is used to check and record the temperature of the refrigerator			
Never	33 (18.6%)	25 (41.0%)	.0001*
Sometimes	125 (70.6%)	24 (39.3%)	
Always	19 (10.7%)	12 (19.7%)	

	Inbuilt thermometer in freezer		
	Present (*n* = 163)	Absent (*n* = 75)	
Thermometer is used to check and record the temperature of the freezer			
Never	25 (15.3%)	33 (44.0%)	.0001*
Sometimes	118 (72.4%)	31 (41.3%)	
Always	20 (12.3%)	11 (14.7%)	

* *p*‐value significant.

A comparison of the practice of monitoring appliance temperature based on the type of restaurant is shown in Table 2. International restaurants had a significantly higher proportion of appliances with built‐in thermometers than local restaurants (*p* = .0001). International restaurants had significantly higher temperature‐monitoring data available for their appliances than local restaurants (88.1% vs 63.3%; *p* = .0001). This difference in the practice may be because of the awareness of international restaurants about the fact that if they are unable to ensure proper food temperature management, their business may have significant financial consequences. However, we found that both international and local restaurants had a poor practice of monitoring and documenting appliance temperatures “always” (16.1% and 10.0%, respectively). Our study findings are in line with the results of studies conducted in other countries (Bolton et al., 2008; Henroid Jr. & Sneed, 2004; Osaili et al., 2013; Walker & Jones, 2002; Youn & Sneed, 2003).

**TABLE 2:** Comparison of presence of inbuilt thermometer in refrigerator and freezer and monitoring practices based on type of restaurants (*n* = 238 restaurants).

	International restaurant (*n* = 118)	Local restaurant (*n* = 120)	*p*‐value
Refrigerator has built‐in thermometer			
Yes	96 (81.4%)	81 (67.5%)	.017*
No	22 (18.6%)	39 (32.5%)	
Thermometer is used to check and record the temperature of the refrigerator			
Never	14 (11.9%)	44 (36.7%)	.0001*
Sometimes	85 (72.0%)	64 (53.3%)	
Always	19 (16.1%)	12 (10.0%)	
Freezer has a built‐in thermometer			
Yes	102 (86.4%)	61 (50.8%)	.0001*
No	16 (13.6%)	59 (49.2%)	
Thermometer is used to check and record the temperature of the freezer			
Never	14 (11.9%)	44 (36.7%)	.0001*
Sometimes	85 (72.0%)	64 (53.3%)	
Always	19 (16.1%)	12 (10.0%)	

* *p*‐value significant.

Usually, the responsibility of monitoring and documenting the temperatures of the appliances lies with the restaurant supervisor or manager and under the general regulations of MOMRA the presence of a qualified manager is not required in Saudi Arabia (Alsayeqh, 2015b). Therefore, in‐job training programs are required for the food handler to educate them about the importance of temperature‐monitoring systems like regular monitoring will help in early identification of any change in appliance temperatures and corrective actions can be initiated to avoid stored food to enter the danger zone temperature. Inefficiency in temperature monitoring would result in safety regulatory compliance violations, food‐borne illness, and unnecessary food wastage. The training should emphasize that these systems play a significant role in building a restaurant culture that is economically, socially, and environmentally more sustainable.

### 3.2 Prevalence of food thermometer in restaurants

The prevalence of food thermometers in restaurants was found to be 53.4% (127/238 restaurants) and all restaurants had an electronic food thermometer. International restaurants compared to local restaurants showed higher prevalence of food thermometer [114/118 (96.6%) restaurants vs. 13/120 (10.8%) restaurants; *p* = .0001]. Food thermometer availability in the restaurant is not a justification for its usage, but it provides an insight that the restaurants are informed about the requirement of the instrument in their business. Studies have reported that undercooked meat is responsible for FBDs due to pathogens such as *Salmonella* serotypes, *E. coli*, and *C. jejuni* (Al‐Mutairi et al., 2015; Boore et al., 2010; USDA, n.d., https://www.fsis.usda.gov/food‐safety/foodborne‐illness‐and‐disease). Our study findings highlight that the local restaurants are at‐risk sites for FBDs as they lack food thermometer, which is the most reliable method to verify food is adequately cooked. The finding suggests that rigorous inspections are needed to be conducted and fines to be charged for lack of functional food thermometer in the establishment. According to Alsayeqh (2015a, 2015b) in KSA there are <2500 health inspectors employed by MOMRA, which have the responsibility to perform inspections of restaurants, food‐sale stores, hotels, and other public health services. The MOMRA should collaborate with Ministry of education (MOE) to initiate academic programs on food safety in colleges offering undergraduate degree in food science and nutrition and to encourage all the universities to start these courses to overcome the shortage in workforce.

### 3.3 Practice of food thermometer usage among the 127 chef participants

**FIGURE 1:**
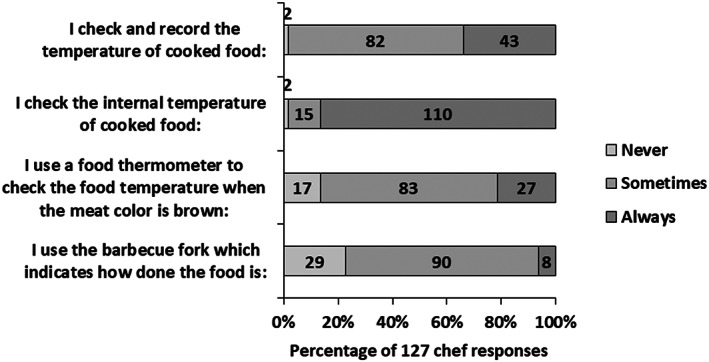
Responses of 127 chefs on use of food thermometer.

The chefs' demographic profile is shown in Table 3 and their responses to the questions on their temperature‐monitoring practices are shown in Figure 1. The majority of the chefs [81 (63.8%)] were in the 21–35 age group, and education level of high school and above [102 (83.3%)]. Ninety (70.9%) chefs reported the use of a barbecue fork “sometimes” to confirm that food was cooked. All chefs responded that they “never” used hands to check whether food was cooked. The practice followed by chefs in international and local restaurants is shown in Table 4. The practice of “always” checking the internal temperature of cooked food was significantly higher for chefs working in international restaurants than for those in local restaurants (90.4% vs. 53.8%, *p* = .0001). Although the practice of using a food thermometer “always” to check the internal temperature of cooked food in international restaurants was high (90.4%), the recording and documentation of the temperature of cooked food were poor (32.5%). Similar findings were reported by the studies conducted in different countries (Henroid, & Sneed, 2004; Mustaffa et al., 2017; Youn & Sneed, 2003; Walker & Jones, 2002).

**TABLE 3:** Demographic details of the participated chefs (*n* = 127 chefs).

	Chef (*n* = 127)	Percentage
Age (years)		
21–35	81	63.8
36–50	46	36.2
Education		
Secondary school	25	16.7
High school or above	102	83.3
Work experience (years)		
<5	50	39.4
5–10	55	43.3
>10	22	17.3

**TABLE 4:** Comparison of food thermometer usage practice among chefs in international and local restaurants (*n* = 127 chefs).

	International restaurant (*n* = 114)	Local restaurant (*n* = 13)	*p*‐value
I use the barbecue fork which indicates how done the food is			
Never	29 (25.4%)	0 (0.0%)	.051
Sometimes	77 (67.5%)	13 (100.0%)	
Always	8 (7.0%)	0 (0.0%)	
I use a food thermometer to check the food temperature when the meat color is brown			
Never	14 (12.3%)	3 (23.1%)	.111
Sometimes	73 (64.0%)	10 (76.9%)	
Always	27 (23.7%)	0 (0.0%)	
I check the internal temperature of cooked food			
Never	2 (1.8%)	0 (0.0%)	.0001*
Sometimes	9 (7.9%)	6 (46.2%)	
Always	103 (90.4%)	7 (53.8%)	
I check and record the temperature of cooked food			
Never	2 (1.8%)	0 (0.0%)	.568
Sometimes	75 (65.7%)	7 (53.8%)	
Always	37 (32.5%)	6 (46.2%)	

* p‐value significant.

Our previous study conducted with restaurant managers revealed that they had a fair‐to‐good level of knowledge about the internal temperature of cooked meat and the best method to check for safe cooking temperature, that is, using a thermometer and a cold food storage temperature (Al‐Mohaithef et al., 2021). Therefore, the employment of a well‐qualified manager in the restaurant can help in effective time temperature control management. An experienced certified manager can motivate the food handlers through appreciation letters or rewards for recording and documenting critical temperatures daily. Furthermore, he may set himself as a good example by monitoring and documenting critical temperature, which may help to bring about a behavioral change among his subordinate food handlers.

A significantly higher percentage of chefs in the 21–35 age group and education level high school and above “always” used a food thermometer to check the food temperature when meat turned brown, compared to their counterparts (28.4% vs. 8.7%, *p* = .007 and 22.6% vs. 16.0%, respectively) (Table 5 and Table 6). Moreover, chef's education level also showed significant association with the practice of using barbecue forks to confirm that food is cooked (*p* = .010) (Table 6). The practice of food thermometer usage did not show a significant difference with work experience (Table 7). A training and education program is required for restaurant managers and other food handlers on proper food handling practices, such as the correct way of measuring food temperature, cleaning and sanitizing of food thermometers to prevent cross‐contamination, and accurate methods to calibrate food thermometers. The program should also include information on how to increase the documentation of critical temperatures, such as the end‐point temperature of prepared food and appliance temperatures. In KSA, the training of restaurant employees was initiated in 2009 in the eastern province of the Saudi Arabia, 1 and by 2013, staff of 171 restaurants out of 440 restaurants were trained and designated as “qualified” (Lin, 2018). However, the effect of this training program has not been assessed to date.

**TABLE 5:** Comparison of food thermometers usage among chefs based on to their age (*n* = 127 chefs).

	Age (years)		*p*‐value
	21–35 (*n* = 81)	36–50 (*n* = 46)	
I use the barbecue fork which indicates how done the food is:			
Never	14 (17.3%)	15 (32.6%)	.133
Sometimes	62 (76.5%)	28 (60.9%)	
Always	5 (6.2%)	3 (6.5%)	
I use a food thermometer to check the food temperature when the meat color is brown:			
Never	13 (16.0%)	4 (8.7%)	.007*
Sometimes	45 (55.6%)	38 (82.6%)	
Always	23 (28.4%)	4 (8.7%)	
I check the internal temperature of cooked food:			
Never	2 (2.5%)	0 (0.0%)	.074
Sometimes	13 (16.0%)	2 (4.3%)	
Always	66 (81.5%)	44 (95.7%)	
I check and record the temperature of cooked food:			
Never	2 (2.5%)	0 (0.0%)	.183
Sometimes	48 (59.3%)	34 (73.9%)	
Always	31 (38.3%)	12 (26.1%)	

* *p*‐value significant.

**TABLE 6:** Comparison of food thermometers usage among chefs based on education (*n* = 127 chefs).

	Education level		*p*‐value
	Secondary school (*n* = 25)	High school or above (*n* = 102)	
I use the barbecue fork which indicates how done the food is:			
Never	4 (16.0%)	25 (24.5%)	.010*
Sometimes	20 (84.0%)	69 (67.7%)	
Always	0 (0.0%)	8 (7.8%)	
I use a food thermometer to check the food temperature when the meat color is brown:			
Never	8 (32.0%)	9 (8.8%)	.009*
Sometimes	13 (52.0%)	70 (68.6%)	
Always	4 (16.0%)	23 (22.6%)	
I check the internal temperature of cooked food:			
Never	0 (0.0%)	2 (2.0%)	.817
Sometimes	4 (16.0%)	11 (10.8%)	
Always	21 (84.0%)	89 (87.2%)	
I check and record the temperature of cooked food:			
Never	0 (0.0%)	2 (2.0%)	.608
Sometimes	15 (60.0%)	67 (65.7%)	
Always	10 (40.0%)	33 (32.3%)	

* *p*‐value significant.

**TABLE 7:** Comparison of food thermometers usage among chefs depending on work experience (*n* = 127 chefs).

	Work experiences (years)			*p*‐value
	<5 (*n* = 50)	5–10 (*n* = 55)	>10 (*n* = 22)	
I use the barbecue fork which indicates how done the food is:				
Never	10 (20.0%)	14 (25.5%)	5 (22.7%)	.545
Sometimes	37 (74.0%)	39 (70.9%)	14 (63.6%)	
Always	3 (6.0%)	2 (3.6%)	3 (13.6%)	
I use a food thermometer to check the food temperature when the meat color is brown:				
Never	6 (12.0%)	9 (16.4%)	2 (9.1%)	.645
Sometimes	31 (62.0%)	35 (63.6%)	17 (77.3%)	
Always	13 (26.0%)	11 (20.0%)	3 (13.6%)	
I check the internal temperature of cooked food				
Never	1 (2.0%)	1 (1.8%)	0 (0.0%)	.814
Sometimes	4 (8.0%)	8 (14.5%)	3 (13.6%)	
Always	45 (90.0%)	46 (83.6%)	19 (86.4%)	
I check and record the temperature of cooked food				
Never	1 (2.0%)	1 (1.8%)	0 (0.0%)	.968
Sometimes	33 (66.0%)	35 (63.6%)	14 (63.6%)	
Always	16 (32.0%)	19 (34.5%)	8 (36.4%)	

### 3.4 Strength and limitation

To the best of our knowledge, this is the first study conducted in Saudi Arabia to assess and validate the practice of refrigerator thermometers. In our study, we measured the temperature of refrigerators and freezers, validated the monitoring practice followed in the restaurant by reviewing the logbook, and also confirmed the presence of a food thermometer in the restaurant before providing the survey link to the chef for its completion. The limitations of the study are as follows. First, the response rate of participation was 68%, which might have resulted in nonresponse bias leading to uncertainty in the representativeness of the study sample. Second, only the temperatures of refrigerator and freezer were examined, and the temperature of stored food was not measured to validate that the stored food was within the range of SFDA guideline temperatures. Third, data collected from chefs regarding their use of food thermometers were based on self‐report, which might have led to a social desirability bias, especially in the higher rate of responses for not using hands to check food is cooked and for checking the internal temperature of cooked food. Last, this study was carried out exclusively in Dammam, so the generalization of results is limited. However, the study provides insight into the use of thermometers in restaurants.

### 3.5 Future area of research

Studies are required to assess and validate the practice of hand hygiene, events of cross‐contamination, and food thermometer usage in restaurant kitchens as these are the main causes leading to FBDs. Further studies should also be conducted to validate the storage conditions of TCS foods, such as cheese, yogurt, salad, raw and cooked eggs, meat, fish, and chicken in the refrigerator in restaurants. Randomized controlled trials should be designed to evaluate the effectiveness of training programs on time temperature control and hygiene practices. More research studies should be conducted, especially in Mecca and Medina, as these are the Holy places of Islam where millions of pilgrims visit throughout year and are dependent on restaurants and other food establishments for their meals.

## 4 CONCLUSION

The study results showed poor monitoring and documentation of refrigerator and freezer temperatures along with a low prevalence of food thermometer use. The study results provide insight into one of the barriers to the implementation of the HACCP system in Dammam.

## FUNDING INFORMATION

None.

## CONFLICT OF INTEREST STATEMENT

We have no conflict of interest to declare.

## Supporting information


Appendix S1.

## Data Availability

Data are available from the corresponding author and can be obtained upon request.
