# Supplementary material for: Prevalence of food thermometers usage and temperature control in restaurants in Dammam, Saudi Arabia
Source: Food Sci Nutr. 2023 Mar 10;11(6):3246–54. doi: 10.1002/fsn3.3305 (PMC10261754; doi:10.1002/fsn3.3305)
Supplement: Supplementary file 1 — Appendix S1. [file FSN3-11-3246-s001.docx]

**Questionnaire 1 (to be filled by researcher)**

1. Type of restaurant: 1- Local 2- International

2. Refrigerator has built-in thermometer: 1- Yes 2- No

If ‘Yes’

a. Check and record the temperature of refrigerator: ________°C

b. Temperature reflecting in display is correct: 1- Yes 2- No

If ‘No’

a. Check and record the temperature of refrigerator: ________°C

b. Temperature in the hanging thermometer is correct: 1- Yes 2- No

3. Temperature of refrigerator recorded in logbook: 1- Yes 2- No

If ‘Yes’, check the logbook for entries:

1-Entries are available for 5 to 7 days in a week – ‘Always’

2- Entries are available for 2 to 4 days in a week – ‘Sometimes’

3-Entries are available for 0 to 1 day in a week – ‘Never’

4. Freezer has built-in thermometer: 1- Yes 2- No

If ‘Yes’

a. Check and record the temperature of freezer: ________°C

b. Temperature reflecting in display is correct: 1- Yes 2- No

If ‘No’

a. Check and record the temperature of freezer: ________°C

b. Temperature is below -180°C: 1- Yes 2- No

5. Temperature of freezer recorded in logbook: 1- Yes 2- No

If ‘Yes’, check the logbook for entries:

1-Entries are available for 5 to 7 days in a week – ‘Always’

2- Entries are available for 2 to 4 days in a week – ‘Sometimes’

3-Entries are available for 0 to 1 day in a week – ‘Never’

6. Restaurant has food thermometer: 1- Yes 2- No

If ‘Yes’

1- Metallic 2- Electronic

**Questionnaire 2: (to be filled by chef)**

***Section 1: Demographics***

1. Type of restaurant: 1. Local 2. International

2. Age: 1. 21-30 years 2. 31-40 years 3. 41-50 years

3. Education: 1. Secondary 2. High School 3. Graduate

4. Work experience: 1. <5 years 2. 5-10 years 3. >10 years

***Section 2: Practice questions***

1. I use the barbecue fork which indicates how done the food is:

1. Never 2. Sometimes 3. Always

2. I use food thermometer to check food temperature when the meat color is brown:

1. Never 2. Sometimes 3. Always

3. I use hands to check whether food was cooked or not

1. Never 2. Sometimes 3. Always

4. I check internal temperature for cooked foods:

1. Never 2. Sometimes 3. Always

5. I check and record the temperature of cooked food:

1. Never 2. Sometimes 3. Always
